# Patterns and correlates of visual impairment and ocular hypertension among older adults in the general Chinese population: results from the CKB Biobank

**DOI:** 10.1136/bjo-2024-326620

**Published:** 2025-08-20

**Authors:** Zilun Shao, Aolin Li, Jun Lv, Canqing Yu, Lang Pan, Pei Pei, Ling Yang, Yiping Chen, Yilei Li, Dan Schmidt, Maxim Barnard, Hubert Lam, Fiona Bragg, Junshi Chen, Zhengming Chen, Liming Li, Huaidong Du, Dianjianyi Sun, The China Kadoorie Biobank Collaborative Group, Junshi Chen

**Affiliations:** 1 Department of Epidemiology and Biostatistics, Peking University School of Public Health, Beijing, China; 2 Peking University Center for Public Health and Epidemic Preparedness & Response, Beijing, China; 3 Clinical Trial Service Unit & Epidemiological Studies Unit (CTSU), Nuffield Department of Population Health, Oxford, UK; 4 NCDs Prevention and Control Department, Meilan CDC, Haikou, Hainan, China; 5 Health Data Research UK Oxford, University of Oxford, Oxford, UK; 6 China National Center for Food Safety Risk Assessment, Beijing, China; 7 China Kadoorie Biobank Collaborative Group, Beijing, China

**Keywords:** Public health, Ocular Hypertension, Glaucoma, Epidemiology, Vision

## Abstract

**Background:**

While numerous ophthalmology-specific cohort studies have been conducted in China, there is a significant lack of comprehensive, population-based study on the potential determinants of visual impairment and ocular hypertension (OHT) in the general Chinese population.

**Methods:**

In the 2020–2021 resurvey of the China Kadoorie Biobank study, ~25 000 randomly selected participants from 10 diverse localities (5 urban and 5 rural) were surveyed. Presenting visual acuity (PVA) was measured using the Random E eye chart and intraocular pressure (IOP) was measured using a handheld Icare (ic100) tonometer. Associations of sociodemographic and other factors with risks of visual impairment and OHT were examined using multivariable logistic regression.

**Results:**

Among the 24 613 (aged 45–95 years, 64.4% women) participants, 21.8% had visual impairment (PVA <0.50) and 18.4% had OHT (IOP >18.6 mm Hg). The prevalence rate of visual impairment increased dramatically with age (49.3% in those ≥75 years vs 8.9% in those <55 years), but an opposite trend was observed for OHT. Risks of these two eye conditions were both inversely associated with household income and fish consumption but positively with systolic blood pressure and blood glucose. Higher education was associated with a higher OHT risk but not with visual impairment. Body mass index was inversely associated with visual impairment but positively with OHT. Age-related macular degeneration and glaucoma were most strongly associated with risk of visual impairment, followed by cataract.

**Conclusion:**

The relatively high prevalence rates of poor vision and OHT in China suggest that well-targeted public health interventions should be developed.

**WHAT IS ALREADY KNOWN ON THIS TOPIC**

- Previous studies have primarily focused on the national prevalence and burden of eye health in China, with very limited reports suggesting potential risk factors for visual impairment or ocular hypertension (OHT) or glaucoma from China. For the few such studies conducted in China, they tended to include participants from a certain region, without a comprehensive assessment of visual impairment and specific eye conditions across multiple urban and rural areas using standardised measurements in general population.

**WHAT THIS STUDY ADDS**

- This is the first study reported potential determinants of visual impairment and intraocular pressure (IOP) across multiple urban and rural areas in China. China Kadoorie Biobank study has collected a large amount of data on demographic, socioeconomic, lifestyle, environmental factors and physical characteristics, enabling comprehensive analysis of potential risk factors associated with eye health. The findings showed relatively high prevalence rates of poor vision and OHT in China. Significant associations were found for advanced age, female sex, education, household income, dietary intake, physical activity, body mass index (BMI), systolic blood pressure and blood glucose with either or both of these two eye conditions. Interestingly, the mean IOP and prevalence of OHT decreased with increasing age in both men and women, which contrasted with findings from Western studies. In addition, BMI was positively associated with visual impairment prevalence but was inversely associated with OHT. Higher education was also associated with a higher prevalence of OHT. Age-related macular degeneration and glaucoma were most strongly associated with risk of visual impairment, followed by cataract.

**HOW THIS STUDY MIGHT AFFECT RESEARCH, PRACTICE OR POLICY**

- These findings provide valuable local evidence for designing public health interventions to improve eye health in China. The novel findings, particularly the inverse association of IOP/OHT with age and BMI, along with its strong positive association with education level, may guide further studies aiming to better understand the aetiology of OHT in Chinese or East Asians.

## Introduction

 Eye health is a significant public health and sustainable development concern, not only affecting educational and economic opportunities but also reducing the quality of life and increasing mortality rates.^1^ Moreover, eye condition is a substantial burden and formidable challenge, as almost everyone will experience at least one eye condition during their lifetime.^2^

As China is rapidly moving towards an ageing society, the burden of eye-related diseases is becoming increasingly heavy. Based on the Global Burden of Disease Study, from 1990 to 2019, China experienced the largest increase in age­-standardised prevalence of moderate visual impairment among the G20 countries and was one of the few G20 countries where the prevalence of severe visual impairment had increased.^3^ Despite that, there is a lack of nationwide data on the prevalence or incidence patterns of visual impairment and common eye diseases among adults in China. Glaucoma is the second leading cause of blindness after cataract and the first leading cause of irreversible visual impairment worldwide (because vision loss caused by cataract can be fully restored through surgery), making it extremely important for eye-related research.^4^ The pathogenesis and aetiology of glaucoma are not fully understood, with intraocular pressure (IOP) being recognised as the only modifiable risk factor for glaucoma.^5^ Therefore, investigating correlates and determinant factors may provide valuable mechanistic insights for glaucoma, helping to improve preventive and therapeutic strategies.

Previous studies in China have primarily been based on ophthalmology-specific cohorts,^6^ resulting in a significant lack of visual impairment and elevated IOP in the general population. Moreover, most risk factors associated with eye health have been established through findings from Western studies,^7^ with limited reliable evidence from China and other developing countries, where sociodemographic characteristics, lifestyle factors and disease patterns differ significantly from those in Western populations. Therefore, we aimed to estimate the prevalence of visual impairment and high IOP status and to explore their potential associations with sociodemographic characteristics, lifestyle factors and eye-related diseases in the China Kadoorie Biobank (CKB) study.

## Methods

### Study design and participants

The CKB is a nationwide prospective cohort study with over 512 000 participants, aged 30–79 years at baseline in 2004–2008. Participants were recruited from 10 geographically diverse (5 rural and 5 urban) areas across China. At local study assessment clinics, trained health workers administered a laptop-based electronic questionnaire recording sociodemographic factors, lifestyle (eg, alcohol drinking, smoking, diet, physical activity) and medical history; undertook physical measurements (eg, blood pressure, anthropometry); and collected a blood sample for long-term storage and on-site measurements. Ethical approval was obtained from local, national and international ethical committees. All participants provided written informed consent. Details of the CKB study design and survey methods have been described previously.^8^

After the completion of baseline survey, we randomly selected 5%–6% of the surviving participants for repeated resurveys every 5–6 years, using procedures similar to those applied at baseline with some additional enhancements. In the current analysis, we used data from the third resurvey of CKB, which was conducted from August 2020 to December 2021. A total of 25 087 adults, aged 45–95 years at the time of resurvey.^9^

### Assessment of visual acuity and IOP

Visual acuity (VA) in both eyes was measured using the Random E vision chart. Participants stood at a distance of 5 metres, covering one eye with a light shield. VA was recorded as the lowest line that the direction of letter Es could be correctly identified. In this study, VA was assessed as presenting visual acuity (PVA), reflecting the participant’s vision as they appear for the examination. For those who normally wear glasses or contact lenses, the measurements were done while they wore their own corrective devices. Furthermore, participants were also asked to report on whether they have been diagnosed with myopia or hyperopia, and the corresponding degree of correction. According to the criteria established by the WHO,^2^ the best eye presenting with VA worse than 6/12 (or 0.50) was defined as visual impairment.

IOP was measured at least six times for each eye (without glasses or contact lenses), using a handheld Icare (ic100) tonometer (Icare Finland Oy). The device reports the mean value of random four satisfactory measurements as the final result of IOP measurement for each eye, and the highest IOP value between the two eyes was recorded as the IOP of a participant. Given that Icare100 measurements were on average 4.2 mm Hg lower than those measured with the Goldman Applanation Tonometer (GAT),^10^ in the current study participants with IOP values higher than 16.8 mm Hg were classified as OHT.

### Collection of other information

Sociodemographic characteristics, lifestyle factors and personal health and medical history were collected using a laptop-based electronic questionnaire.^8^ Total physical activity amount in MET-hours per day was calculated using the method reported previously.^11^ The diagnosis and treatment history of major eye diseases, including glaucoma, cataract, age-related macular degeneration (AMD), myopia and hyperopia, was also collected during questionnaire survey. Physical measures and blood glucose were collected using calibrated instruments and following standard procedures. Body mass index (BMI) was calculated as weight (kg) divided by the square of standing height (m).

### Statistical analysis

From the 25 087 CKB participants who participated in the third resurvey, we first excluded 174 who did not complete the questionnaire survey, then excluded 300 incomplete eye-related measurements, leaving a total of 24 613 participants in the current analysis.

Baseline characteristics were described as mean (SD) for continuous variables or percentage for categorical variables by sex, with adjustment for age and region as appropriate, by means of either multiple linear regression or logistic regression. Standardised prevalence rates of visual impairment and OHT were calculated with adjustment for age, sex and region as appropriate. The trend of prevalence by covariables was tested using the Cochran-Armitage test.

Logistic regression analyses were used to explore the associations of visual impairment (better eye PVA <0.50) and OHT status with potential correlates including sociodemographic and lifestyle factors as well as physical measurements and self-reported history of eye diseases. Linear regression analyses were also performed to examine the associations of PVA and IOP with these potential correlates. Stepwise approach was adopted to determine the covariates to be included in the analytical models. Additionally, we performed tests for linear trends for multiple-level categorical variables. In sensitivity analysis, we excluded participants with a self-reported history of these conditions or related treatments. All statistical analyses were conducted using R V.4.0.3, and all p values were two-sided.

## Results

Among the 24 613 participants included in the study, the mean age was 64.9 years, 64.4% were women, and 40.9% were from urban areas (table 1). The overall mean PVA was 0.62, with women having a slightly lower value than men. About 21.9% participants were classified as visually impaired. The overall mean IOP was 13.6 mm Hg, slightly higher in women than in men. A total of 18.4% participants had OHT, slightly higher in women than in men. PVA and IOP were only mildly correlated with each other (r=0.02, p<0.001), similar in men and women (r=0.04 vs 0.02). Both PVA and IOP showed general consistency between the left and right eyes, with very minor differences across the 10 regions (online supplemental eFigure 1 and 2).

**Table 1:** Characteristics of study participants by sex*

Characteristics	Men	Women	Total
Number of participants, n (%)	**8770 (35.6)**	**15 843** (**64.4**)	**24 613**
Age† (years)	65.7 (9.3)	64.5 (9.0)	64.9 (9.1)
Region, n (% of urban):	3462 (39.2)	6595 (41.8)	10 057 (40.9)
Highest education, n (%):			
No formal school	614 (6.3)	3594 (23.7)	4208 (17.1)
Primary school	3140 (34.5)	5387 (34.8)	8527 (34.6)
Middle/high school	4503 (53.7)	6285 (38.4)	10 788 (43.8)
College degree or above	513 (6.4)	577 (3.5)	1090 (4.4)
Annual household income, n (%):			
<10 000 yuan	706 (7.5)	1328 (8.7)	2034 (8.3)
10 000–50 000 yuan	2408 (27.1)	4729 (30.1)	7137 (29.0)
50 000–1 00 000 yuan	2741 (32.2)	5146 (32.0)	7887 (32.0)
>100 000 yuan	2915 (33.3)	4640 (29.3)	7555 (30.7)
Fresh fruits, n (%):			
Monthly or never/rarely	2275 (24.6)	2866 (18.7)	5141 (20.9)
>1 day per week	3629 (40.5)	5831 (37.3)	9460 (38.4)
Daily	2866 (34.8)	7146 (43.9)	10 012 (40.7)
Fish, n (%):			
Never/rarely	2957 (32.9)	6066 (38.7)	9023 (36.7)
Monthly	1677 (19.3)	2743 (17.2)	4420 (18.0)
>1 day per week	4136 (47.8)	7034 (44.1)	11 170 (45.4)
Red meat, n (%):			
Monthly or never/rarely	1516 (16.6)	3551 (22.9)	5067 (20.6)
>1 day per week	4082 (45.8)	7026 (44.8)	11 108 (45.1)
Daily	3172 (37.9)	5266 (32.4)	8438 (34.3)
Physical activity† (MET-h/d)	17.6 (15.8)	13.9 (11.9)	15.2 (13.5)
Body mass index† (kg/m^2^)	24.3 (3.3)	24.5 (3.6)	24.4 (3.5)
Systolic blood pressure† (mm Hg)	132.3 (20.5)	133.6 (21.4)	133.0 (21.1)
Blood glucose† (mmol/L)	6.5 (2.6)	6.6 (2.6)	6.5 (2.6)
Presenting visual acuity†	0.68 (0.32)	0.59 (0.30)	0.62 (0.31)
Visual acuity status, n (%):			
Normal	7201 (83.1)	12 039 (75.2)	19 240 (78.2)
Mild impairment‡	1013 (11.0)	2380 (15.4)	3393 (13.8)
Moderate–severe impairment§	539 (5.7)	1375 (9.0)	1914 (7.8)
Blindness¶	17 (0.2)	49 (0.3)	66 (0.3)
Intraocular pressure†, mm Hg	13.5 (3.6)	13.7 (3.6)	13.6 (3.6)
Ocular hypertension**, n (%)	1562 (17.6)	2965 (18.9)	4527 (18.4)
Self-reported eye diseases, n (%)			
Glaucoma	78 (0.9)	208 (1.3)	286 (1.2)
Cataract	907 (9.8)	2361 (15.3)	3268 (13.3)
Age-related macular degeneration	79 (0.9)	194 (1.2)	273 (1.1)
Hyperopia	30 (0.3)	60 (0.4)	90 (0.4)
Myopia	116 (1.4)	229 (1.4)	345 (1.4)

* Data were adjusted for age, and region where appropriate.

† Values represent mean (SD).

‡ Defined as better eye visual acuity range from 0.30 to 0.50.

§ Defined as better eye visual acuity from 0.05 to 0.30.

¶ Defined as better eye visual acuity<0.05.

** Defined as the highest IOP value between the two eyes >16.8 mm Hg.

IOP, intraocular pressure; MET-h/d, metabolic equivalents of task per hours per day.

### PVA and prevalence of visual impairment by age, sex and region

**Figure 1:**
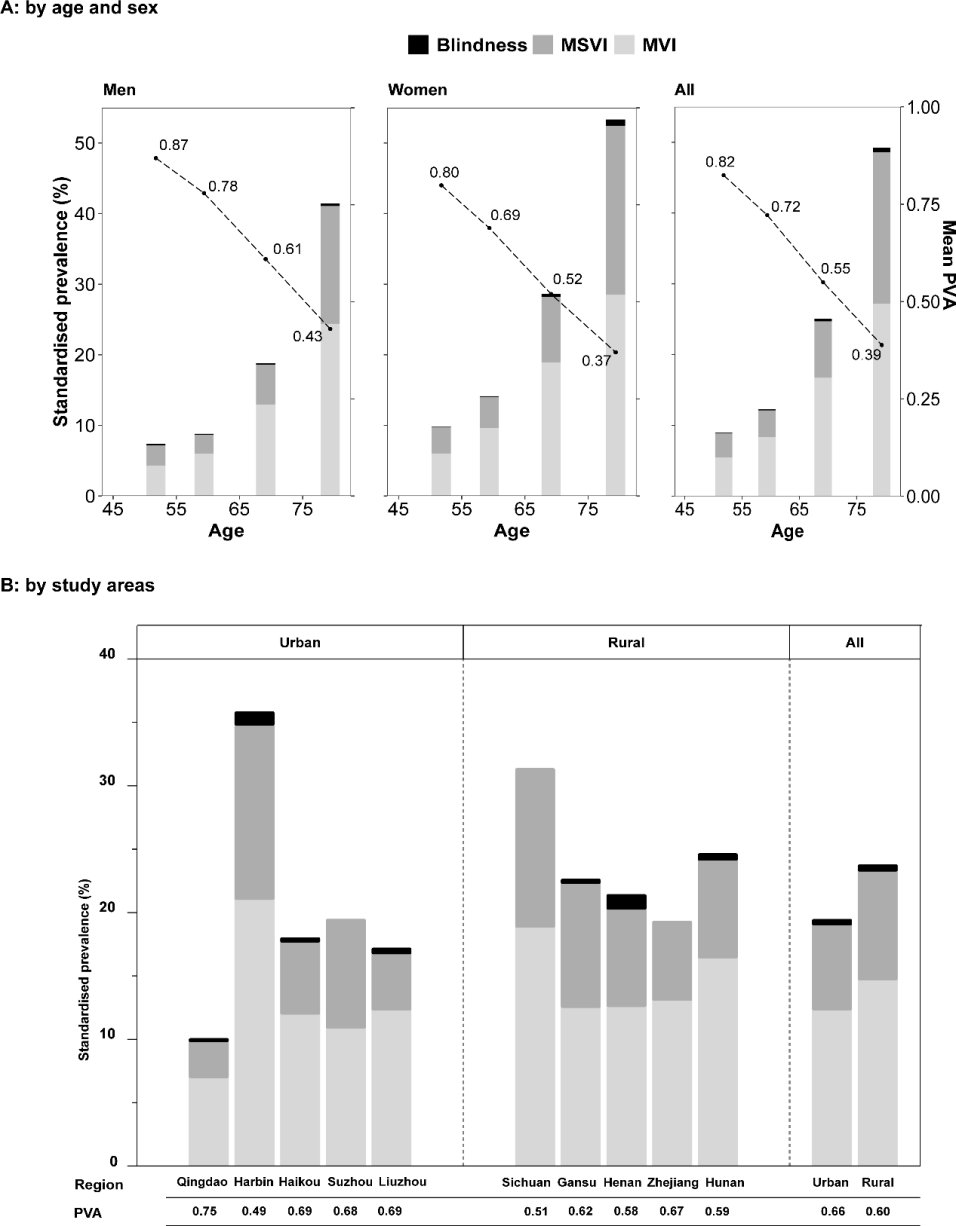
Prevalence of visual impairment prevalence and mean visual acuity by sex, age (A) and study areas (B). Adjusted for age, sex and study areas where appropriate. MVI, defined as the better eye PVA between 0.30 and 0.50; MSVI, defined as PVA between 0.05 and 0.30; Blindness, defined as PVA <0.05. (A) shared the same y-axis, the left y-axis was the standardised prevalence (represented by the bars), and the right y-axis was the mean visual acuity (represented by the dots). P for trend<0.001 (A). MVI, mild visual impairment; MSVI, moderate-severe visual impairment; PVA, presenting visual acuity.

As expected, PVA greatly decreased and the prevalence of visual impairment greatly increased with age, with the lowest mean PVA (0.39) and the highest visual impairment prevalence (49.3%) observed in the oldest age group (≥75 years), in contrast to the 0.82 and 8.9% in the youngest age group (< 55 years old) (figure 1A). Across all age categories, women had a lower PVA than men and a higher prevalence of visual impairment. This pattern was presented in both younger and older groups, as shown in online supplemental eTable 1. As shown in figure 1B, urban participants on average had higher PVA (0.66 vs 0.60) and lower prevalence of visual impairment (19.4% vs 23.7%) than those from rural areas, except for Harbin, an urban region in Northern China with the highest incidence rate of cardiometabolic diseases among the 10 CKB regions,^12^ where the lowest mean PVA (0.49) and highest prevalence was observed (35.7%).

### IOP and prevalence of OHT by age, sex and region

**Figure 2:**
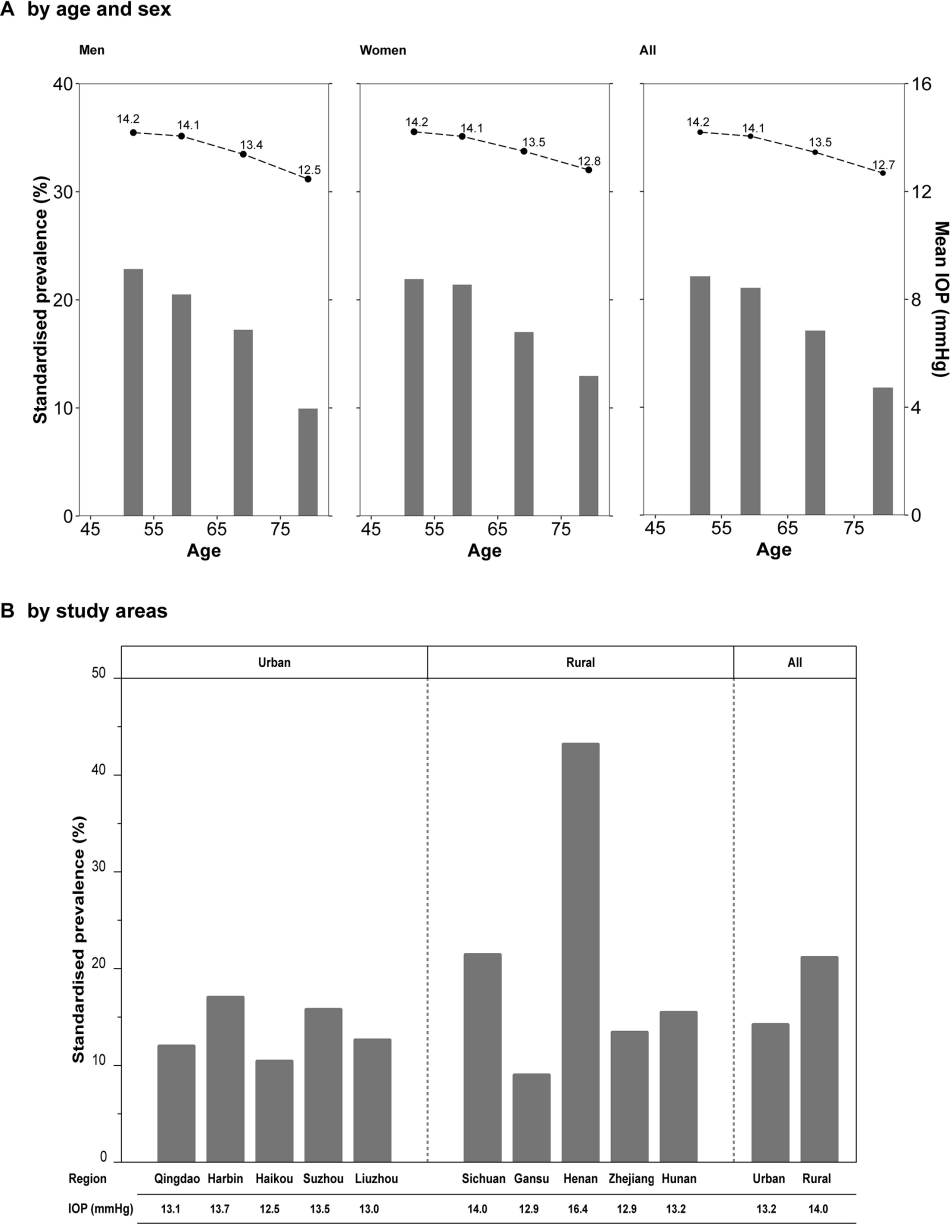
Prevalence of ocular hypertension and mean intraocular pressure by sex, age (A) and study areas (B). Adjusted for IOP seasonal variation and age, sex and study areas where appropriate. (A) shared the same y-axis, the left y-axis was the standardised prevalence (represented by the bars) and the right y-axis was the mean IOP (represented by the dots). P for trend <0.001 (A). IOP, intraocular pressure.

In contrast to the increasing prevalence of visual impairment with age, the mean IOP and prevalence of OHT decreased with increasing age in both men and women (figure 2A), a trend that also persisted after excluding participants with self-reported glaucoma. The mean IOP was about 1.7 mm Hg and 1.4 mm Hg, respectively, lower for men and women in the older age groups than those in the younger age groups, while the corresponding difference in OHT prevalence was even larger. In men, the prevalence of OHT reduced over 50%, from 22.8% in the youngest to 9.9% in the oldest age group. In women, a similar nearly 50% reduction (from 21.9% to 13.0%) was observed. In addition, although the mean IOP values were very similar in urban and rural areas, the prevalence of OHT was significantly higher in rural areas than in urban areas (figure 2B). This rural–urban difference was driven by the two rural regions, Sichuan and Henan. Henan had the highest prevalence of OHT (43.0%) and mean IOP (16.4 mm Hg) among the 10 regions, followed by Sichuan where the corresponding values were 21.7% and 14.0 mm Hg, respectively.

### Socioeconomic, lifestyle and physical correlates of PVA and visual impairment

Household income, but not education, was significantly associated with PVA and risk of visual impairment. Comparing to those with lowest annual household income, participants in the highest category had 0.07 higher PVA (0.63 vs 0.56) and 33% lower risk of visual impairment (table 2 and online supplemental eTable 3).

**Table 2:** Multiple adjusted ORs of visual impairment and ocular hypertension according to sociodemographic characteristics, lifestyle factors and physical measurements*

Subgroups	Visual impairment			Ocular hypertension		
	Number of cases	OR (95% CI)	P	Number of cases	OR (95% CI)	P
Region			0.48			<0.001
Urban	2035	1.00 (Ref)		1390	1.00 (Ref)	
Rural	3338	1.06 (0.97 to 1.16)		3137	1.26 (1.15 to 1.39)	
Highest education			<0.001			<0.001
No formal school	1425	1.00 (Ref)		590	1.00 (Ref)	
Primary school	2162	0.85 (0.78 to 0.93)		1511	1.17 (1.04 to 1.31)	
Middle/high school	1588	0.72 (0.64 to 0.79)		2252	1.41 (1.25 to 1.58)	
College degree or above	198	1.04 (0.85 to 1.27)		174	1.53 (1.23 to 1.90)	
Annual household income (Yuan RMB)			<0.001			0.52
<10 000	716	1.00 (Ref)		412	1.00 (Ref)	
10 000–50,000	1721	0.87 (0.77 to 0.98)		1332	0.86 (0.75 to 0.99)	
50 000–1 00 000	1597	0.76 (0.67 to 0.87)		1556	0.94 (0.82 to 1.08)	
>100 000	1339	0.67 (0.59 to 0.76)		1227	0.73 (0.63 to 0.84)	
Alcohol consumption						
Never regular	3900	1.00 (Ref)	0.87	3495	1.00 (Ref)	0.02
Occasional	668	0.85 (0.77 to 0.94)		835	0.95 (0.86 to 1.04)	
Ex-regular	307	1.10 (0.94 to 1.29)		282	1.12 (0.95 to 1.32)	
Weekly	498	0.99 (0.87 to 1.13)		743	1.17 (1.04 to 1.33)	
Smoking						
Never smoker	4045	1.00 (Ref)	0.51	3813	1.00 (Ref)	0.10
Occasional	203	1.29 (1.07 to 1.56)		198	0.92 (0.76 to 1.11)	
Ex-regular	406	1.11 (0.95 to 1.30)		379	0.97 (0.83 to 1.15)	
Smoker	719	1.06 (0.93 to 1.21)		965	1.10 (0.97 to 1.26)	
Fresh fruits			0.01			0.002
Monthly or never/rarely	1430	1.00 (Ref)		1011	1.00 (Ref)	
>1 day per week	1976	0.87 (0.80 to 0.95)		1843	0.96 (0.87 to 1.05)	
Daily	1967	0.90 (0.82 to 0.99)		1673	0.94 (0.85 to 1.04)	
Per 100 g		0.98 (0.95 to 1.00)			0.99 (0.96 to 1.02)	
Fish			<0.001			<0.001
Never/rarely	2354	1.00 (Ref)		2077	1.00 (Ref)	
Monthly	1004	1.00 (0.91 to 1.10)		869	0.83 (0.75 to 0.91)	
>1 day per week	2015	0.75 (0.69 to 0.83)		1581	0.66 (0.60 to 0.72)	
Per 50 g		0.89 (0.83 to 0.95)			0.85 (0.79 to 0.91)	
Red meat			<0.001			0.03
Monthly or never/rarely	1274	1.00 (Ref)		763	1.00 (Ref)	
>1 day per week	2285	1.04 (0.95 to 1.14)		2288	1.21 (1.09 to 1.34)	
Daily	1814	1.19 (1.08 to 1.31)		1476	0.99 (0.89 to 1.11)	
Per 50 g		1.08 (1.05 to 1.12)			0.94 (0.90 to 0.97)	
Physical activity†			0.001			0.04
Q1	1838	1.00 (Ref)		1036	1.00 (Ref)	
Q2	1429	0.86 (0.79 to 0.94)		1129	0.99 (0.90 to 1.10)	
Q3	1212	0.83 (0.76 to 0.92)		1182	1.00 (0.91 to 1.11)	
Q4	894	0.86 (0.77 to 0.95)		1180	0.89 (0.80 to 0.99)	
Per 4 MET-h/d		0.98 (0.96 to 0.99)			0.99 (0.98 to 1.00)	
BMI (kg/m^2^)			<0.001			<0.001
<18.5	250	1.13 (0.95 to 1.35)		72	0.68 (0.52 to 0.88)	
18.5–24	2529	1.00 (Ref)		1690	1.00 (Ref)	
24–28	1842	0.82 (0.76 to 0.88)		1887	1.21 (1.12 to 1.31)	
≥28	752	0.86 (0.77 to 0.95)		878	1.48 (1.34 to 1.64)	
Per 1 kg/m^2^		0.97 (0.96 to 0.98)			1.05 (1.04 to 1.06)	
Systolic blood pressure (mm Hg)			<0.001			<0.001
<120	1207	1.00 (Ref)		892	1.00 (Ref)	
120–140	1835	1.10 (1.00 to 1.20)		1585	1.46 (1.33 to 1.60)	
140–160	1333	1.10 (1.00 to 1.22)		1204	1.76 (1.59 to 1.95)	
≥160	776	1.14 (1.02 to 1.28)		724	2.40 (2.13 to 2.70)	
Per 10 mm Hg		1.02 (1.01 to 1.04)			1.15 (1.13 to 1.17)	
Blood glucose (mmol/L)			<0.001			<0.001
<5.0	1013	0.95 (0.87 to 1.04)		758	0.86 (0.78 to 0.95)	
5.0–7.0	2768	1.00 (Ref)		2401	1.00 (Ref)	
7.0–9.0	801	1.04 (0.94 to 1.14)		759	1.17 (1.06 to 1.29)	
9.0–11.0	290	1.16 (1.00 to 1.35)		232	1.07 (0.91 to 1.25)	
≥11.0	434	1.39 (1.22 to 1.58)		357	1.36 (1.18 to 1.55)	
Per 1 mmol/L		1.04 (1.02 to 1.05)			1.04 (1.02 to 1.05)	

Visual impairment status was redefined into two categories (visual impairment and non-visual impairment).

Analysis was adjusted for age, sex, region, education, household income, alcohol consumption, smoking, fresh fruits, fish, red meat, physical activity, BMI, systolic blood pressure, blood glucose and seasonal variation (only for OHT).

* P values for multiple-level categorical variables were based on linear trend tests.

† The quartiles of physical activity were determined separately for men and women. The cut-off values were 5.60, 11.70 and 25.60 for men, and 5.63, 10.29 and 17.99 for women.

BMI, body mass index; MET-h/d, metabolic equivalents of task per hour per day; OHT, ocular hypertension; Ref, reference.

Among the lifestyle factors, although smoking and alcohol consumption were not in a clear significant association with PVA or risk of visual impairment, several dietary factors and physical activity seemed relevant for vision. Weekly fish consumption (>1 day per week) was associated with 25% lower risk of visual impairment than those never fish consumers; daily red meat consumption was associated with 19% higher risk than those with consumption level lower than one per week; and daily fresh fruit consumption was related to 10% lower risk than those either never/rarely or only monthly consume fresh fruit. Consistently, these lifestyle factors were also significantly associated with PVA (online supplemental eTable 2). Highest (sex-specific) quartile of physical activity was associated with 14% lower risk (OR=0.86, 95% CI 0.77 to 0.95) of visual impairment than those in the bottom quartile group, although the linear relationship between the two was only borderline significant.

Higher BMI was significantly associated with a lower risk of visual impairment, with each 1-unit higher BMI associated with an OR of 0.97 for visual impairment (table 2) and a 0.002 increment of adjusted PVA (online supplemental eTable 2). Compared with those with BMI between 18.5 kg/m^2^ and 24 kg/m^2^ (ie, normal weight^13^), the OR for visual impairment was 0.86 for those with BMI at least 28 kg/m^2^. Although BMI and SBP were positively correlated with each other (r=0.13), SBP was in a strong positive association with PVA before and after adjusting for BMI. For each 10 mm Hg higher SBP, the adjusted mean value of PVA was 0.004 lower and the risk of visual impairment was 1.02 higher (online supplemental eTable 2 and table 2). A similar positive association was observed for blood glucose, with each 1 mmol/L higher being associated with 0.004 lower PVA (online supplemental eTable 2) and 4% higher risk of visual impairment (table 2). Compared with those having random blood glucose between 5.0 mmol/L and 7.0 mmol/L, the OR for visual impairment was 1.39 among those with blood glucose levels of at least 11.0 mmol/L (table 2).

### Socioeconomic, lifestyle and physical correlates of IOP and OHT

Education, but not household income, was in a clear positive association with IOP and risk of OHT (table 2). Compared with those with no formal education, those with a college degree or above had 53% higher risk of OHT. No clear significant association was observed between smoking and alcohol consumption and IOP, but weekly alcohol drinking was associated with a significantly higher risk of OHT than never drinkers, with OR of 1.17. Among those three dietary variables who were significantly associated with visual impairment, only fish consumption showed a significant association with IOP and risk of OHT. Compared to those who never/rarely consume fish, those with at least once per week consumption having 34% lower risk of OHT 0.66, with each 50 g daily consumption associated with OR of 0.85.

There was a clear linear positive association of BMI, SBP and blood glucose with IOP and risk of OHT (online supplemental eTable 2 and 3). Each 1 kg/m^2^ higher BMI, 10 mm Hg higher SBP and 1 mmol/L blood glucose were associated with 0.09 mm Hg, 0.27 mm Hg and 0.07 mm Hg, respectively, higher IOP. Compared with those who were normal weight, those who were obese had 48% higher risk of OHT (OR=1.48, 1.34 to 1.64) while those with BMI <18.5 kg/m^2^ had 32% lower risk of OHT (OR=0.68, 0.52 to 0.88). Across categories of SBP, OR for OHT prevalence increased significantly from 1.46 (1.33–1.60) and 1.76 (1.59–1.95) to 2.40 (2.13–2.70), respectively, in contrast to those with SBP <120 mm Hg as reference group (p for trend <0.001). The prevalence risk for OHT in those with blood glucose above or equal to 11.0 mmol/L was 36% higher than that in participants with blood glucose between 5.0 mmol/L and 7.0 mmol/L.

### Associations of self-reported eye diseases with visual impairment and OHT

**Figure 3:**
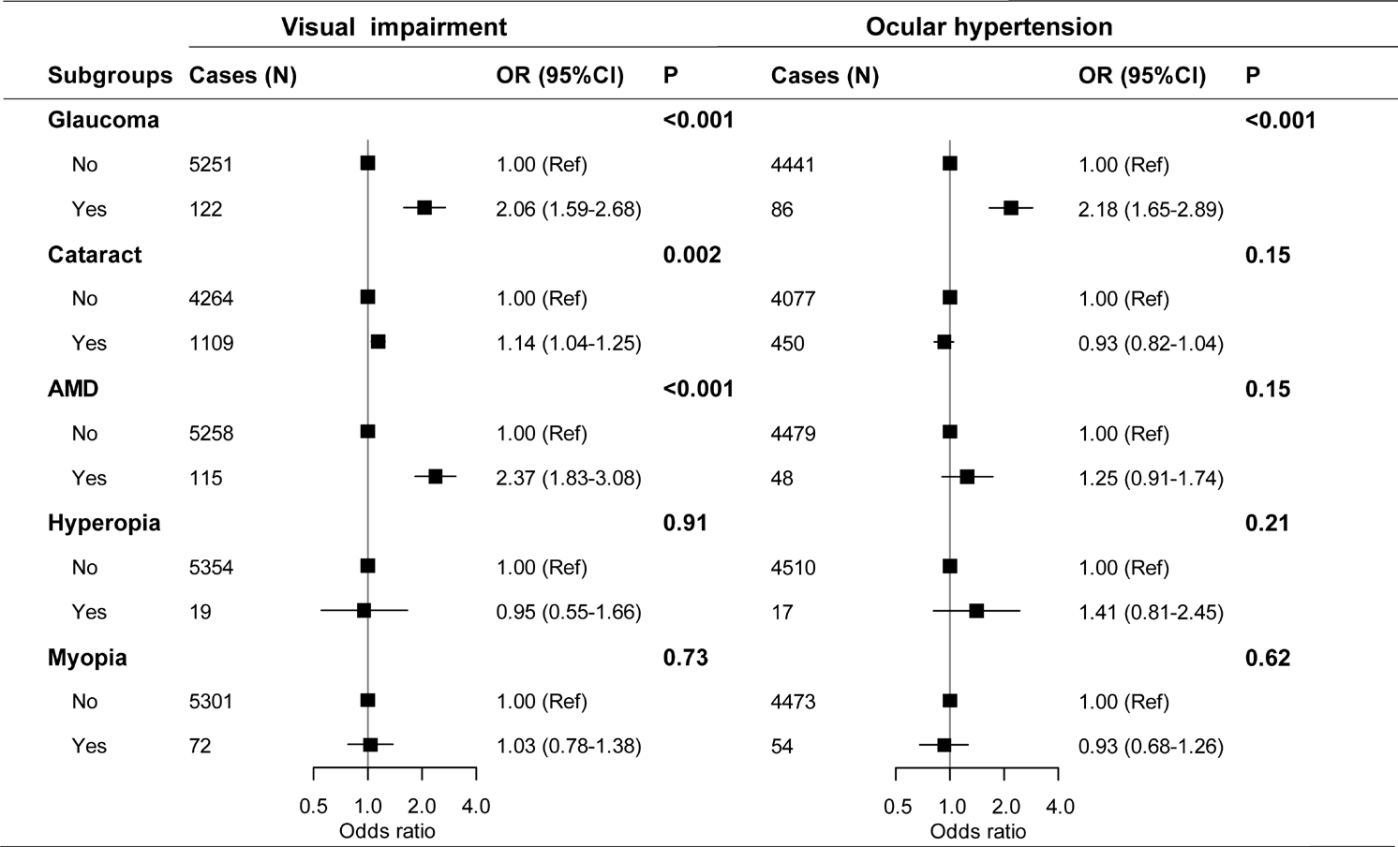
Multiple adjusted ORs of visual impairment and ocular hypertension according to self-reported history of eye diseases. Visual impairment status was redefined into two categories (visual impairment and non-visual impairment). Analysis was adjusted for age, sex, region, education, household income, alcohol consumption, smoking, fresh fruits, fish, red meat, physical activity, BMI, systolic blood pressure, blood glucose, seasonal variation (only for OHT) and eye diseases (glaucoma, cataract, AMD, myopia, hyperopia). AMD, age-related macular degeneration; BMI, body mass index; OHT, ocular hypertension.

As expected, AMD, glaucoma and cataract were all associated with poorer vision (online supplemental eTable 3) and higher risk of visual impairment, with AMD having the largest OR (2.37, 1.83–3.08), followed by glaucoma (2.06, 1.59–2.68) and cataract (1.14, 1.04–1.25) (figure 3). Those who have reported a history of glaucoma also had a 1.65 mm Hg higher IOP on average and more than two times higher risk of OHT compared with those without such history (OR=2.18, 1.65 to 2.89). No significant associations were observed for cataract, AMD, myopia and hyperopia with IOP or risk of OHT (online supplemental eTable 3 and figure 3).

## Discussion

In this relatively large population-based survey, we found that poor vision was associated with an older age, female sex, lower level of household income, lower intake of fresh fruit and fish and higher intake of red meat, lower level of physical activity, lower BMI, elevated SBP and higher levels of blood glucose. No clear geographical pattern of visual impairment was observed, despite the highest prevalence found in that Harbin, an urban city in the Northern China with the highest incidence of cardiometabolic diseases among the 10 CKB regions.^12^ In contrast, OHT had a completely opposite association with age and BMI. The risk of having OHT was about half (OR=0.38) in the oldest group than those in the youngest group and was nearly 50% higher (OR=1.48) in those with BMI ≥28 kg/m^2^ compared with those BMI between 18.5 and 24 kg/m^2^. In addition, although education level was not in a clear linear relationship with vision, it was in a highly significant positive association with OHT, with those having a college or higher degree had more than 50% higher risk than those without formal education. Among the self-reported eye diseases investigated, only glaucoma was significantly related to a higher risk of OHT, but AMD, glaucoma and cataract were all associated with a poorer vision. We conducted sensitivity analyses by excluding participants with a self-reported history of these conditions or related treatments and found nearly identical results.

Despite the strong positive association between age and visual impairment, which is under expectation,^2^ the inverse association between age and IOP/OHT observed in our study is unexpected. The majority of previous findings from Western studies support a higher risk of OHT in older age groups,^14 15^ but some studies in East Asian populations, such as Japanese and Chinese populations, demonstrated either an inverse relationship^16^,^18^ or no association.^19^ In our study, IOP was measured using a rebound tonometer rather than a GAT, which may have influenced the measurements. However, two previous Japanese population-based studies, both using GAT to measure IOP, also reported a significant inverse association between IOP and age.^16 18^ One possible explanation for this might be that the production of aqueous humour may decrease with age.^20^ Additionally, the corneal biomechanical properties may explain this trend. With ageing, there is a general thinning of the cornea, which correlates with lower IOP measurements.^21^ Therefore, this trend might be caused by difference in corneal thickness. Nevertheless, several previous studies also found a similar inverse relationship between age and IOP after adjusting for corneal thickness and hysteresis,^22 23^ suggesting that other age-related physiological mechanisms may also be involved in influencing IOP.

The sex pattern of visual impairment and OHT observed in our study was consistent across 10 regions and with previous findings in different populations. For example, after controlling for age, global estimates suggested that approximately 7% more women than men present with impairment of distance vision.^2^ Another small study based on older Swedish adults showed that the mean IOP in women was on average 1.22 mm Hg higher than in men.^24^ Although the reasons for this observed sex difference are incompletely understood, it could reflect a variety of factors. First, pregnancy can cause vision-related diseases such as diabetic retinopathy, vascular occlusions, serous retinal detachments and central serous retinopathy.^25^ Second, women may have more limited access to healthcare services than men, particularly in developing areas.^1^ Third, oestrogens may regulate IOP by influencing the aqueous production and outflow systems,^26^ and progesterone may increase the resistance to aqueous outflow and thereby cause a rise in IOP.

Although not entirely consistent, visual impairment and OHT seemed to be associated with lower SES defined by income level in the current study, in agreement with previous findings.^7^ But no previous study has reported a similar positive association between length of education and OHT as observed in the current study. However, our results fit in the well-established previous evidence supporting that there is a direct association between longer education and myopia^27^ and between myopia and elevated IOP and glaucoma.^28^ This suggests that near vision tasks might contribute to OHT and potentially increase the risk of glaucoma. Further investigation through randomised controlled trials is needed to establish the causality of the observed association.

Our study provides some evidence on the role of a healthy lifestyle for eye health. These findings are consistent with previous studies conducted in Western populations. Fresh fruits are rich in antioxidant vitamins, which have the potential to reduce the risk of various eye conditions, including cataract, glaucoma, diabetic retinopathy and AMD,^29^ which are among the main causes of visual impairment.^2^ Vitamin C is highly concentrated in human lens and aqueous humour and has been associated with ocular ascorbate levels, which is believed to protect the lens against photo-oxidative damage.^30^ Omega-3 fatty acids, which are found in fish, are important for proper visual development and retinal function, and an adequate intake of these fatty acids through the diet is essential for optimal visual development.^31^ Meta-analyses of large-scale studies have demonstrated that a higher intake of omega-3 fatty acids or fish is associated with a lower risk of AMD, which is a main contributor of poor vision in Western communities.^2^ Consistent with our findings, a large number of studies have shown that increased consumption of red meat is associated with a higher risk of AMD.^32^ This may be attributed to the higher levels of nitrosamines or N-nitroso compounds, heme iron and advanced glycation end products that are found in red meat.^33^ Moreover, red meat is abundant in omega-6 fatty acids, which compete with omega-3 fatty acids for the same metabolic enzymes. Elevated red meat consumption diminishes the protective effects of omega-3 fatty acids on eye health, consequently elevating the risk of eye diseases.

Proven benefits of physical activity on the nervous system, coupled with the shared embryonic origins of many ocular components with the central nervous system, suggest the plausibility of the inverse association between physical activity and PVA observed in the current study.^34^ Although previous studies have reported immediate or short-term decreases in IOP after exercise, suggesting that physical activity may have a beneficial effect on IOP,^34^ our study did not find a clear association between physical activity and IOP.

The positive associations of SBP and blood glucose with visual impairment and OHT were in agreement with previous evidence,^14 35^ supporting the important role of cardiometabolic health on vision and eye health. In addition, blood pressure also has a dynamic role in aqueous production as well as in regulating aqueous outflow by the effect on episcleral venous pressure and the pulse-dependent motion of the trabecular meshwork, therefore dramatically influencing IOP and OHT risk. The positive association of BMI with OHT is under-expected because of the close relationship of BMI with SBP and blood glucose. However, the exact reasons for the moderate inverse association between BMI and visual impairment are unknown, with reverse causality being one possible explanation because those with poor vision may develop malnutrition in the longer term.

Finally, among the self-reported eye diseases investigated, AMD and glaucoma had the strongest associations with visual impairment, followed by cataract. This is in line with the fact that AMD and glaucoma, once in advanced stage and influencing vision, are irreversible but cataract could be treated to recover patients’ sight.

Our study has several strengths, including a relatively large number of participants recruited from multiple rural and urban regions in China, and a comprehensive assessment of various factors related to vision and eye health. However, several limitations should be considered as well. First, our study population is a random sample of the surviving participants in the CKB study, not representative nationally or of each specific participating region in China. Second, we did not have individuals younger than 45 years, therefore limiting our ability to provide information on younger populations. Furthermore, as mentioned above, our study did not collect data on corneal biomechanics, which could be the potential underlying factor link age and lower values of IOP. Although this is a recognised limitation, several previous studies also found a similar inverse relationship between age and IOP after adjusting for corneal thickness and hysteresis,^22 23^ suggesting that other age-related physiological mechanisms may also be involved in influencing IOP. Moreover, we did not perform detailed refractive error tests and information on prior history of eye diseases, particularly myopia and hyperopia, was solely self-reported, limiting our opportunities to perform separate analyses among participants with or without these diseases. Another significant limitation of our study is the reliance on self-reported eye diseases, which may lead to non-differential misclassification bias, but the result is a bias towards the null. Finally, the cross-sectional design of our study restricts our ability to control reverse causality, and the observational nature of the study suggests that residual confounding may exist despite the adjustment for a wide range of potential confounders.

## Conclusion

In this largest study to date on the prevalence patterns and potential determinants of visual impairment and OHT among older adults in the general Chinese population, we found that visual impairment and OHT share some, but not complete, similarities in their associations with sociodemographic, lifestyle and health-related factors. These findings provide valuable local evidence for designing public health interventions to improve eye health in China. In addition, novel findings from the current study (eg, the inverse association of IOP/OHT with age and the strong positive association between OHT and education level) also highlight the need for further studies to better understand the aetiology of specific eye diseases in Chinese or East Asians. However, given the cross-sectional design and observational nature of our study, the findings should not be directly interpreted as a causal relationship or having direct clinical implications for individual diagnosis or screening. Rather, they highlight patterns at the population level that underscore further investigation through longitudinal and clinical research.

## Supplementary material

10.1136/bjo-2024-326620 online supplemental file 2

## Data Availability

Data are available upon reasonable request.
