# Supplementary material for: Patterns and correlates of visual impairment and ocular hypertension among older adults in the general Chinese population: results from the CKB Biobank
Source: Br J Ophthalmol. 2025 Aug 20;110(1):e326620. doi: 10.1136/bjo-2024-326620 (PMC7618444; doi:10.1136/bjo-2024-326620)
Supplement: online supplemental file 2 [file bjo-110-1-s001.docx]

# Patterns and correlates of visual impairment and ocular hypertension among older adults in the general Chinese population: results from the CKB Biobank

# SUPPLEMENTARY MATERIALS

**eFigure 1:** Violin plot showing the distribution of visual acuity in all participants and each CKB region

**eFigure 2:** Violin plot showing the distribution of IOP in all participants and each CKB region

**eTable 1:** Prevalence and values of visual impairment and ocular hypertension by age group (≤65 and >65)

**eTable 2:** Multiple linear regression results for continuous variables

**eTable 3:** Least-squares means for continuous outcomes

**Members of the China Kadoorie Biobank collaborative group**

## eFigure 1: Violin plot showing the distribution of visual acuity in all participants and each CKB region


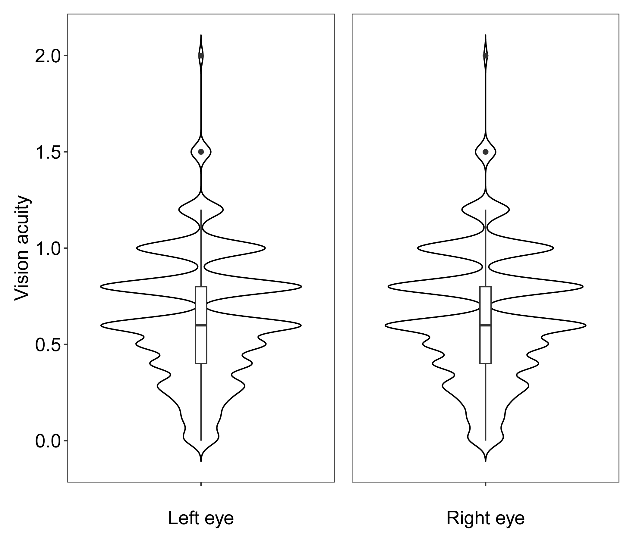


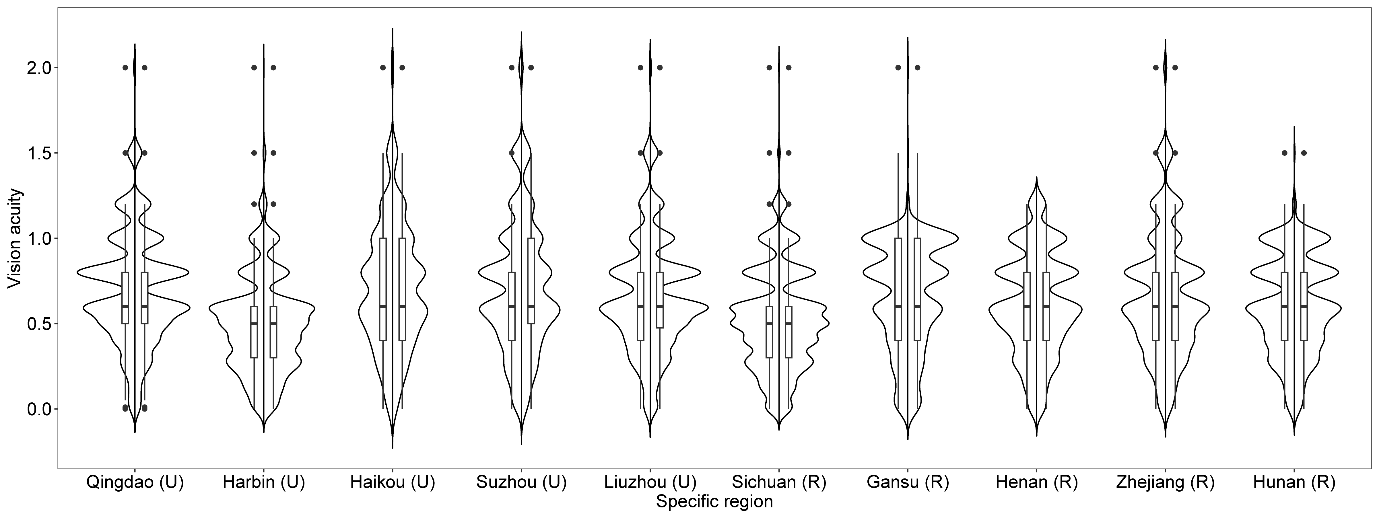


^a^ In specific region plots, the left part of each region shows left eye visual acuity distribution and the right part shows right eye visual acuity distribution.

## eFigure 2: Violin plot showing the distribution of mean IOP in all participants and each CKB region


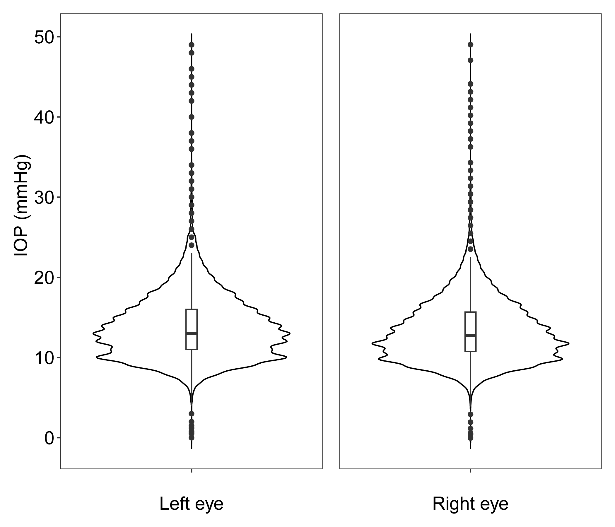


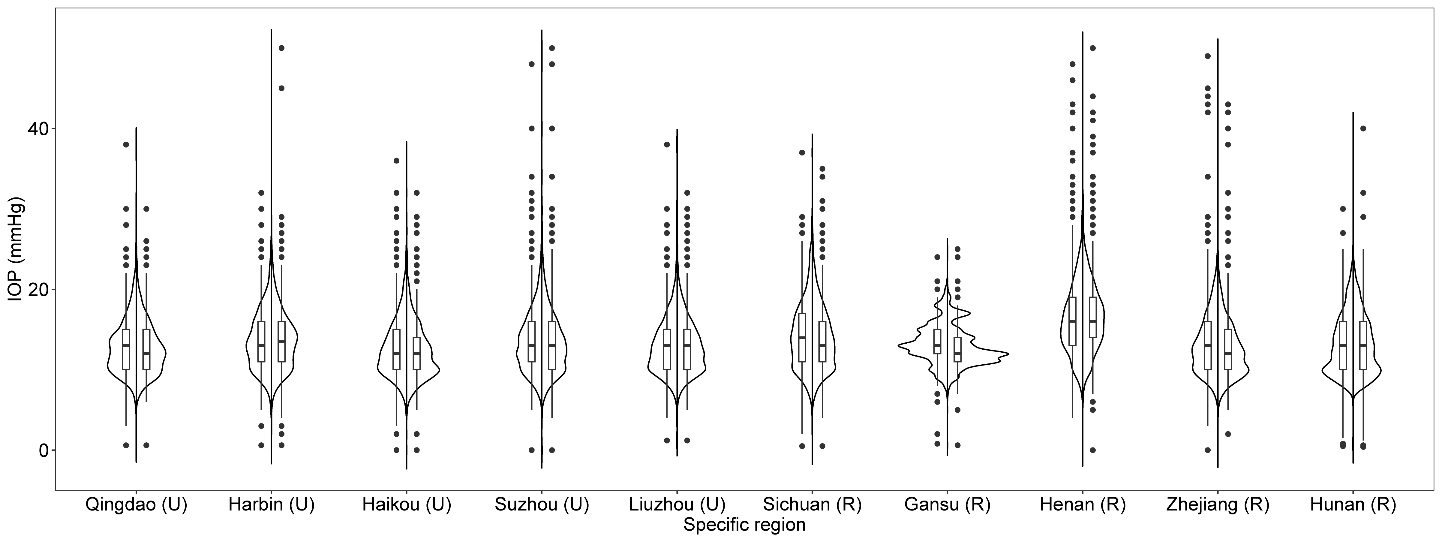


^a^ In specific region plots, the left part of each region shows left eye mean IOP distribution and the right part shows right mean IOP distribution.

## eTable 1: Prevalence and values of visual impairment and ocular hypertension by age group (≤65 and >65)

|  | **Men** | **Women** | **All** |
| --- | --- | --- | --- |
| **Visual impairment (%)^a^** |  |  |  |
| Age group |  |  |  |
| ≤65 | 8.8 | 13.5 | 11.9 |
| >65 | 26.7 | 36.0 | 32.5 |
| **Visual acuity** |  |  |  |
| Age group |  |  |  |
| ≤65 | 0.79 | 0.71 | 0.74 |
| >65 | 0.55 | 0.47 | 0.50 |
| **Ocular hypertension (%)^b^** |  |  |  |
| Age group |  |  |  |
| ≤65 | 21.5 | 21.6 | 21.5 |
| >65 | 14.3 | 15.4 | 15.0 |
| **IOP (mmHg)** |  |  |  |
| Age group |  |  |  |
| ≤65 | 14.1 | 14.1 | 14.1 |
| >65 | 13.0 | 13.2 | 13.1 |

^a^ Prevalence of visual impairment;

^b^ Prevalence of Ocular hypertension.

## eTable 2: Multiple linear regression results for continuous variables^a^

| **Variables** | **β** | **95% CI** | **P** |
| --- | --- | --- | --- |
| **Visual acuity** |  |  |  |
| Age (year) | -0.0138 | (-0.0143, -0.0134) | <0.001 |
| BMI (kg/m^2^) | 0.0024 | (0.0014, 0.0034) | <0.001 |
| SBP (per 10 mmHg) | -0.0035 | (-0.0052, -0.0018) | <0.001 |
| Blood glucose (mmol/L) | -0.0042 | (-0.0055, -0.0029) | <0.001 |
| **IOP (mmHg)** |  |  |  |
| Age (year) | -0.0728 | (-0.0787, -0.0669) | <0.001 |
| BMI (kg/m^2^) | 0.0943 | (0.0815, 1.0712) | <0.001 |
| SBP (per 10 mmHg) | 0.2696 | (0.2478, 0.2915) | <0.001 |
| Blood glucose (mmol/L) | 0.0717 | (0.0546, 0.0889) | <0.001 |

^a^ Analysis was adjusted for age, sex, region, education, household income, alcohol consumption, smoking, fresh fruits, fish, red meat, physical activity, BMI, systolic blood pressure, blood glucose and seasonal variation (only for OHT).

## eTable 3: Least-squares means for continuous outcomes^a^

| **Variables** | | **N** | **Lsmeans** | **95% CI** | **P^b^** |
| --- | --- | --- | --- | --- | --- |
|  | **Visual acuity** | | | | |
| **Age groups (years)** | |  |  |  | <0.001 |
| 45-54 | | 3694 | 0.74 | (0.73-0.76) |  |
| 55-64 | | 8115 | 0.66 | (0.65-0.67) |  |
| 65-74 | | 8979 | 0.53 | (0.52-0.54) |  |
| ≥75 | | 3825 | 0.93 | (0.38-0.40) |  |
| **Highest education** | |  |  |  | <0.001 |
| No formal school | | 4208 | 0.57 | (0.56-0.58) |  |
| Primary school | | 8527 | 0.60 | (0.59-0.60) |  |
| Middle/high school | | 10788 | 0.63 | (0.62-0.64) |  |
| College degree or above | | 1090 | 0.56 | (0.54-0.58) |  |
| **Annual household income (RMB)** | |  |  |  | <0.001 |
| <10,000 | | 2034 | 0.56 | (0.55-0.58) |  |
| 10,000–50,000 | | 7137 | 0.58 | (0.57-0.59) |  |
| 50,000–100,000 | | 7887 | 0.59 | (0.58-0.60) |  |
| >100,000 | | 7555 | 0.63 | (0.62-0.64) |  |
| **Fresh fruits** | |  |  |  | <0.001 |
| Low | | 5141 | 0.58 | (0.57-0.59) |  |
| Moderate | | 9460 | 0.59 | (0.58-0.60) |  |
| High | | 10012 | 0.59 | (0.59-0.60) |  |
| **Fish** | |  |  |  | <0.001 |
| Low | | 9023 | 0.57 | (0.56-0.58) |  |
| Moderate | | 4420 | 0.58 | (0.56-0.59) |  |
| High | | 11170 | 0.62 | (0.61-0.63) |  |
| **Red meat** | |  |  |  | <0.001 |
| Low | | 5067 | 0.61 | (0.60-0.62) |  |
| Moderate | | 11108 | 0.59 | (0.58-0.60) |  |
| High | | 8438 | 0.57 | (0.56-0.58) |  |
| **BMI (kg/m^2^)** | |  |  |  | <0.001 |
| <18.5 | | 10617 | 0.59 | (0.58-0.60) |  |
| 18.5-24.0 | | 795 | 0.56 | (0.54-0.58) |  |
| 24.0-28.0 | | 9602 | 0.60 | (0.60-0.61) |  |
| >=28.0 | | 3599 | 0.60 | (0.59-0.61) |  |
| **Physical activity** | |  |  |  | <0.001 |
| Q1 | | 6212 | 0.57 | (0.56-0.58) |  |
| Q2 | | 6108 | 0.59 | (0.58-0.60) |  |
| Q3 | | 6139 | 0.60 | (0.59-0.61) |  |
| Q4 | | 6154 | 0.60 | (0.59-0.61) |  |
| **SBP (mmHg)** | |  |  |  | <0.001 |
| <120 | | 6832 | 0.60 | (0.59-0.61) |  |
| 120-140 | | 8445 | 0.59 | (0.58-0.60) |  |
| 140-160 | | 5445 | 0.59 | (0.58-0.60) |  |
| >=160 | | 2687 | 0.58 | (0.57-0.59) |  |
| **Blood glucose (mmol/L)** | |  |  |  | <0.001 |
| <5.0 | | 13148 | 0.60 | (0.59-0.61) |  |
| 5.0-7.0 | | 5032 | 0.60 | (0.59-0.61) |  |
| 7.0-9.0 | | 3555 | 0.60 | (0.58-0.61) |  |
| 9.0-11.0 | | 1188 | 0.58 | (0.57-0.60) |  |
| >=11.0 | | 1490 | 0.56 | (0.55-0.58) |  |
| **Eye diseases** | |  |  |  |  |
| **Glaucoma** | |  |  |  | <0.001 |
| Yes | | 286 | 0.47 | (0.44-0.50) |  |
| No | | 24327 | 0.61 | (0.61-0.62) |  |
| **Cataract** | |  |  |  | 0.002 |
| Yes | | 3268 | 0.57 | (0.56-0.58) |  |
| No | | 21345 | 0.61 | (0.61-0.62) |  |
| **AMD** | |  |  |  | <0.001 |
| Yes | | 273 | 0.43 | (0.40-0.46) |  |
| No | | 24340 | 0.61 | (0.60-0.62) |  |
| **Myopia** | |  |  |  | 0.91 |
| Yes | | 90 | 0.58 | (0.52-0.64) |  |
| No | | 24523 | 0.61 | (0.60-0.61) |  |
| **Hyperopia** | |  |  |  | 0.73 |
| Yes | | 345 | 0.59 | (0.57-0.62) |  |
| No | | 24268 | 0.61 | (0.60-0.61) |  |
|  | **IOP (mmHg)** | | | | |
| **Age groups (years)** | |  |  |  | <0.001 |
| 45-54 | | 3694 | 14.6 | (14.4-14.7) |  |
| 55-64 | | 8115 | 14.2 | (14.1-14.4) |  |
| 65-74 | | 8979 | 13.5 | (13.4-13.7) |  |
| ≥75 | | 3825 | 12.7 | (12.5-12.8) |  |
| **Highest education** | |  |  |  | <0.001 |
| No formal school | | 4208 | 13.5 | (13.3-13.6) |  |
| Primary school | | 8527 | 13.7 | (13.6-13.9) |  |
| Middle/high school | | 10788 | 14.0 | (13.9-14.1) |  |
| College degree or above | | 1090 | 14.2 | (14.0-14.4) |  |
| **Annual household income (RMB)** | |  |  |  | <0.001 |
| <10,000 | | 2034 | 14.1 | (13.9-14.3) |  |
| 10,000–50,000 | | 7137 | 13.8 | (13.7-14.0) |  |
| 50,000–100,000 | | 7887 | 14.0 | (13.9-14.1) |  |
| >100,000 | | 7555 | 13.5 | (13.4-13.6) |  |
| **Fresh fruits** | |  |  |  | 0.001 |
| Low | | 5141 | 13.9 | (13.8-14.1) |  |
| Moderate | | 9460 | 13.9 | (13.8-14.0) |  |
| High | | 10012 | 13.8 | (13.6-13.9) |  |
| **Fish** | |  |  |  | <0.001 |
| Low | | 9023 | 14.2 | (14.1-14.3) |  |
| Moderate | | 4420 | 13.8 | (13.7-14.0) |  |
| High | | 11170 | 13.5 | (13.4-13.7) |  |
| **Red meat** | |  |  |  | 0.04 |
| Low | | 5067 | 13.7 | (13.6-13.9) |  |
| Moderate | | 11108 | 14.1 | (14.0-14.2) |  |
| High | | 8438 | 13.7 | (13.6-13.9) |  |
| **BMI (kg/m^2^)** | |  |  |  | <0.001 |
| <18.5 | | 10617 | 13.7 | (13.6-13.8) |  |
| 18.5-24.0 | | 795 | 13.1 | (12.8-13.4) |  |
| 24.0-28.0 | | 9602 | 14.1 | (14.0-14.2) |  |
| >=28.0 | | 3599 | 14.5 | (14.3-14.6) |  |
| **Physical activity** | |  |  |  | 0.04 |
| Q1 | | 6212 | 13.9 | (13.8-14.0) |  |
| Q2 | | 6108 | 13.9 | (13.8-14.0) |  |
| Q3 | | 6139 | 13.9 | (13.8-14.0) |  |
| Q4 | | 6154 | 13.7 | (13.6-13.8) |  |
| **SBP (mmHg)** | |  |  |  | <0.001 |
| <120 | | 6832 | 13.0 | (12.8-13.1) |  |
| 120-140 | | 8445 | 13.7 | (13.6-13.8) |  |
| 140-160 | | 5445 | 14.1 | (14.0-14.2) |  |
| >=160 | | 2687 | 14.7 | (14.5-14.8) |  |
| **Blood glucose (mmol/L)** | |  |  |  | <0.001 |
| <5.0 | | 13148 | 13.7 | (13.6-13.8) |  |
| 5.0-7.0 | | 5032 | 13.4 | (13.3-13.5) |  |
| 7.0=9.0 | | 3555 | 14.0 | (13.8-14,1) |  |
| 9.0-11.0 | | 1188 | 14.0 | (13.8-14.2) |  |
| >=11.0 | | 1490 | 14.2 | (14.0-14.4) |  |
| **Eye diseases** | |  |  |  |  |
| **Glaucoma** | |  |  |  | <0.001 |
| Yes | | 286 | 15.3 | (14.9-15.7) |  |
| No | | 24327 | 13.7 | (13.6-13.8) |  |
| **Cataract** | |  |  |  | 0.15 |
| Yes | | 3268 | 13.7 | (13.6-13.8) |  |
| No | | 21345 | 13.7 | (13.6-13.8) |  |
| **AMD** | |  |  |  | 0.15 |
| Yes | | 273 | 13.8 | (13.4-14.2) |  |
| No | | 24340 | 13.7 | (13.6-13.8) |  |
| **Myopia** | |  |  |  | 0.21 |
| Yes | | 90 | 14.1 | (13.3-14.8) |  |
| No | | 24523 | 13.7 | (13.6-13.8) |  |
| **Hyperopia** | |  |  |  | 0.62 |
| Yes | | 345 | 13.4 | (13.0-13.7) |  |
| No | | 24268 | 13.7 | (13.6-13.8) |  |

^a^ Analysis was adjusted for age, sex, region, education, household income, alcohol consumption, smoking, fresh fruits, fish, red meat, physical activity, BMI, systolic blood pressure, blood glucose, eye diseases and seasonal variation (only for OHT).

^b^ P values for multiple-level categorical variables were based on linear trend tests.

## Members of the China Kadoorie Biobank collaborative group

**International Steering Committee:** Junshi Chen, Zhengming Chen (PI), Robert Clarke, Rory Collins, Liming Li (PI), Jun Lv, Richard Peto, Robin Walters.

**International Co-ordinating Centre, Oxford:** Daniel Avery, Maxim Barnard, Derrick Bennett, Lazaros Belbasis, Ruth Boxall, Ka Hung Chan, Yiping Chen, Zhengming Chen, Charlotte Clarke, Johnathan Clarke; Robert Clarke, Huaidong Du, Ahmed Edris Mohamed, Hannah Fry, Simon Gilbert, Pek Kei Im, Andri Iona, Maria Kakkoura, Christiana Kartsonaki, Hubert Lam, Kuang Lin, James Liu, Mohsen Mazidi, Iona Millwood, Sam Morris, Qunhua Nie, Alfred Pozarickij, Maryanm Rahmati, Paul Ryder, Saredo Said, Dan Schmidt, Becky Stevens, Iain Turnbull, Robin Walters, Baihan Wang, Lin Wang, Neil Wright, Ling Yang, Xiaoming Yang, Pang Yao.

**National Co-ordinating Centre, Beijing:** Xiao Han, Can Hou, Chao Liu, Jun Lv, Lang Pan, Pei Pei, Dianjianyi Sun, Qingmei Xia, Canqing Yu.

**10 Regional Co-ordinating Centres:**

**Qingdao CDC:** Zengchang Pang, Ruqin Gao, Shanpeng Li, Haiping Duan, Shaojie Wang, Yongmei Liu, Ranran Du, Yajing Zang, Liang Cheng, Xiaocao Tian, Hua Zhang, Yaoming Zhai, Feng Ning, Xiaohui Sun, Feifei Li. **Licang CDC:** Silu Lv, Junzheng Wang, Wei Hou. **Heilongjiang Provincial CDC:** Wei Sun, Shichun Yan, Xiaoming Cui. **Nangang CDC:** Chi Wang, Zhenyuan Wu,Yanjie Li, Quan Kang. **Hainan Provincial CDC:** Huiming Luo, Tingting Ou. **Meilan CDC:** Xiangyang Zheng, Zhendong Guo, Shukuan Wu, Yilei Li, Huimei Li. **Jiangsu Provincial CDC:** Ming Wu, Yonglin Zhou, Jinyi Zhou, Ran Tao, Jie Yang, Jian Su. **Suzhou CDC:** Fang Liu, Jun Zhang, Yihe Hu, Yan Lu, Liangcai Ma, Aiyu Tang, Shuo Zhang, Jianrong Jin, Jingchao Liu. **Guangxi Provincial CDC:** Mei Lin, Zhenzhen Lu. **Liuzhou CDC:** Lifang Zhou, Changping Xie, Jian Lan,Tingping Zhu,Yun Liu, Liuping Wei, Liyuan Zhou, Ningyu Chen, Yulu Qin, Sisi Wang. **Sichuan Provincial CDC:** Xianping Wu, Ningmei Zhang, Xiaofang Chen, Xiaoyu Chang. **Pengzhou CDC:** Mingqiang Yuan, Xia Wu, Xiaofang Chen, Wei Jiang, Jiaqiu Liu, Qiang Sun. **Gansu Provincial CDC:** Faqing Chen, Xiaolan Ren, Caixia Dong. **Maiji CDC:** Hui Zhang, Enke Mao, Xiaoping Wang, Tao Wang, Xi zhang. **Henan Provincial CDC:** Kai Kang, Shixian Feng, Huizi Tian, Lei Fan. **Huixian CDC:** XiaoLin Li, Huarong Sun, Pan He, Xukui Zhang. **Zhejiang Provincial CDC:** Min Yu, Ruying Hu, Hao Wang. **Tongxiang CDC**: Xiaoyi Zhang, Yuan Cao, Kaixu Xie, Lingli Chen, Dun Shen. **Hunan Provincial CDC:** Xiaojun Li, Donghui Jin, Li Yin, Huilin Liu, Zhongxi Fu. **Liuyang CDC:** Xin Xu, Hao Zhang, Jianwei Chen,Yuan Peng, Libo Zhang, Chan Qu.
